# Review of sensory systems deployed by epidermal keratinocytes

**DOI:** 10.3389/fcell.2025.1598326

**Published:** 2025-06-02

**Authors:** Mitsuhiro Denda, Peter M. Elias

**Affiliations:** ^1^ Meiji Institute for Advanced Study of Mathematical Sciences, Meiji University, Nakano-Ku, Japan; ^2^ Department of Dermatology, Northern California Institute for Research and Education, and Veterans Affairs Health Care System, University of California San Francisco, San Francisco, CA, United States

**Keywords:** keratinocyte, epidermis, sensory receptor, barrier homeostasis, central nervous system, pH

## Abstract

Recent studies have shown that epidermal sensory receptors intercept and direct responses to potentially threatening environmental factors, including shifts in temperature, electric potential, sound, acidity, light, taste, and odor. In addition to stimulating epidermal responses, activation of keratinocytes by these stressors can directly signal the central nervous system. Changes in epidermal permeability barrier homeostasis also depend upon ion dynamics, particularly alterations in intraepidermal gradients of calcium (Ca^2+^) and pH. The purpose of this review is to update readers about recent advances in the field of cutaneous sensory receptors, focusing upon their roles in mediating not only permeability barrier function, but also whole-body physiology and certain aspects of mental status.

## 1 Introduction

Maintenance of epidermal permeability barrier homeostasis is the most essential of mammalian cutaneous functions in humans, because a compromised water barrier inevitably triggers inflammatory dermatoses as well as potentially leading to dehydration and even death, as shown in patients with extensive burns and blistering disorders. Epidermal keratinocytes are the epithelial cells of mammalian skin. In the basal layer of the epidermis, these cells proliferate avidly, and as they move outwards towards the skin surface, epidermal differentiation proceeds. In the uppermost layer of the viable epidermis, keratinocytes undergo physiologic apoptosis, forming a thin, water-impermeable layer called the stratum corneum. The stratum corneum, which, is composed of two components, i.e., protein-enriched nonviable cells and extracellular lipid domains. The extracellular lipid enriched lamellar membranes develop following the secretion of myriad lamellar bodies from stratum granulosum cells. Hence, immediately after acute barrier disruption, regardless of whether the specific insult results from organic solvents, detergent applications, or mechanical insults, normal epidermis mounts an immediate, lamellar body secretory response leading to permeability barrier normalization (Grubauer et al., 1989). Hence, the epidermis works diligently to restore optimal function when challenged by diverse environmental stressors.

In normal skin, after acute disruption, the permeability barrier recovers swiftly. However, with repeated or sustained abrogations, inflammatory responses occur (Denda et al., 1996). Thus, prevention of the barrier dysfunction is very important for cutaneous pathology.

The state of barrier homeostasis is linked to a gradient of calcium ions that peaks in the outer, nucleated layers of the epidermis (Lee et al., 1992; Menon et al., 1994), which in turn, regulates epidermal terminal differentiation, surface potential, and lipid secretion. We hypothesized therefore that epidermal keratinocytes possess cation-sensitive, electrical sensory systems that protect against environmental stressors. Pertinently, nerve transmissions similarly rely upon the electrochemical behavior of neurons in the central nervous system. We hypothesized accordingly that the epidermis, which develops in concert with the nervous system from a primitive ectodermal layer that envelopes the developing embryo, could also be influenced by electrochemical gradients. Hence, we evaluated changes in skin surface electric potential in an *ex vivo*, hairless mouse organ culture system, following exposure to either exogenous calcium or calcium ionophores (Denda et al., 2001a) (results are summarized in Table 1).

**TABLE 1:** Alterations IN SKIN surface electrical potential.

Application	Biochemical impact	Electric potential	Effect on impact on barrier recovery
Sodium azide 5%	Induces cell death	Decreases	NYP
Ouabain 50 M	Inhibits Na^+^K^+^ATPase	Decreases	NYP
Trifluoperazine 50 M	Inhibits Ca^2+^ Mg^2+^ ATPase	Decreases	NYP
Amitripyline 50 M	Inhibits Ca^2+^ Mg^2+^ ATPase	Decreases	NYP
Verapamil 50 M	Blocks Ca^2+^ channel	Decreases	Accelerates
Nifedipine 50 M	Blocks Ca^2+^ channel	Decreases	Accelerates
4-aminopyridine 50 M	Blocks K^+^ channel	Decreases	NYP
Tetrodotoxin 50 M	Blocks Na^+^ channel	Decreases	NYP
Ionomycin 50 M	Calcium specific ionophore	Decreases	Delays
Valinomycin 50 M	Potassium ionophore	Decreases	Accelerates
A23187 50 M	Calcium, magnesium ionophore	No effect	NYP
EGTA 2 mM	Chelates calcium	Decreases	NYP
EDTA 2 mM	Chelates calcium and magnesium	No effect	NYP
CRF 0.5 M	Corticotropin releasing factor	Decreases	NYP
+antagonist 0.5 M		No effect	NYP
Substance P 0.5 M	Neurotransmitter	Decreases	NYP
+antagonist 0.5 M		No effect	NYP
Barrier disruption		Decreases	
+ Verapamil 50 M		No effect	Accelerates
+ 4-aminopyridine 50 M		No effect	NYP
+ Tetrodotoxin 50 M		Decreases	NYP

Denda et al. (2001a), Denda et al. (2006), Denda et al. (2007b).

NYP, experiment not yet performed.

Importantly, the epidermis is an active endocrine tissue–it not only generates a variety of biomodulators, that include cortisol releasing factor (CRF), cortisol, opioids, cannabinoids, substance P, oxytocin (OT), thyroid hormone, and melatonin but also receptors for these mediators (Vukelic et al., 2011; Denda et al., 2012a; Takei et al., 2013; Vidali et al., 2016; Slominski et al., 2022; Datta et al., 2022; Samra et al., 2023). For example, the negative consequences of psychological stress on both barrier homeostasis and antimicrobial peptide production are inhibited by both the CRF antagonist, antalarmin, as well as the glucocorticoid receptor antagonist, mifepristone (Ru-486) (Denda et al., 2000; Aberg et al., 2007). Together, these results suggest that a series of hormones and their receptors could regulate epidermal permeability barrier homeostasis and likely antimicrobial defense. In the following sections, we will update current knowledge about how activation of these receptors impacts epidermal structure, function, metabolism, as well as whole body physiology.

## 2 Epidermal sensory systems that respond to physical factors

### 2.1 Temperature

The epidermis expresses a series of transient receptor potential receptors (TRPs), which localize to the upper layer of the epidermis where they monitor and direct responses to fluctuations in potentially threatening environmental stressors. Epidermal TRPV1 is activated by heat (>42°C) and by alterations in acidity, as well as the ‘hot’ chemical ingredient, capsaicin (Caterina et al., 1997). TRPV2 is activated by still higher temperatures (>52°C), while TRPV3 instead is activated at cooler temperatures, as well as by ‘cooling’ chemicals like menthol and camphor. TRPM8 is activated at still lower temperatures (<22°C), as well as by menthol. TRPA1 is activated at even lower temperatures (<17°C), and by several different types of chemical substances (Dhaka et al., 2006). Finally, TRPV4 is activated by warm temperatures, as well as by the receptor agonist, 4αPDD (Watanabe et al., 2002).

We and others demonstrated that these TRPs not only localize to the outer epidermis, but some also regulate permeability barrier homeostasis (Denda et al., 2001b; Inoue et al., 2002; Peier et al., 2002; Chung et al., 2003; Atoyan et al., 2009; Biro and Kovacs, 2009; Tsutsumi et al., 2010; Tsutsumi et al., 2011). For example, activation of TRPV1 by either short exposures to 42°C, or by topical applications of capsaicin delays barrier recovery after acute disruption in mouse epidermis. In contrast, activation of TRPV4 by short exposures to 36–40°C, or by topical application of 4αPDD accelerate recovery in mouse epidermis (Denda et al., 2007a). Likewise, activation of TRPM8 by short exposures to 10–15°C, or by topical applications of menthol accelerate barrier recovery in mouse epidermis (Denda et al., 2010a). Finally, activation of TRPA1 by short exposures to 10–15°C, or by topical application of a TRPA1 agonist also accelerate recovery in mouse epidermis (Denda et al., 2010b). This series of studies indicate that epidermal keratinocytes deploy a variety of sensory systems that respond to challenges from potential external physical factors, which in turn protect permeability barrier homeostasis. Yet, how this system itself is regulated, and the mechanisms by which receptor activation enhances barrier function remain unknown.

The function of each TRP channel might have a variety of activators. For example, previous review indicated that TPPV1 function is regulated by several endogenous factors (Shuba, 2021). Moreover, a recent study demonstrated that TRPV4 forms a complex structure with Rho GTPase, and that interactions with RhoA influenced TRPV4-madiated calcium homeostasis (Kwon et al., 2023).

Though a large number of studies have been demonstrated both epidermal and neural TRP, most have focused on neural sensation, like pain sensation. Recently, by using cryoelectron microscopy, the structure of TRP channels has been partially clarified (Zhang et al., 2021). TRPs have six transmembrane spanning domains, with a pore-forming loop whose structure resembles voltage-gated ion channels (Huang et al., 2024). However, how these thermosensitive mechanisms operate has not yet been clarified. Moreover, recent review indicated that TRPs are permeable to a variety of cations such as Ca^2+^,Mg^2+^,Na^+^, and K^+^ (Zhang et al., 2023). We previously demonstrated that influx of Ca^2+^ into epidermal keratinocytes delays the barrier recovery (Denda et al., 2003b), while influx of K^+^ instead accelerates recovery (Denda et al., 2007b).

### 2.2 Electric potential

Once again, the common origin of the epidermis and the nervous system prompted us to compare the impact of positive and negative electric potential on barrier recovery after acute perturbations. While applications of a positive potential delayed barrier recovery, negative potentials instead accelerate barrier recovery in mouse epidermis (Denda and Kumazawa, 2002). Moreover, applications of either cationic polymers (Denda et al., 2005a), or barium sulfate (Fuziwara et al., 2004), with its negative zeta-potential, accelerate recovery in mouse epidermis. This series of studies indicate that epidermis likely possesses a still uncharacterized sensory system that recognizes and responds to changes in electric potential (Denda, 2005b).

Increase in intracellular calcium ions induce exocytosis of neuromediators at synapses. Analogously, when we applied external negative electric potential onto the surface of the skin, elevations of intracellular calcium and exocytosis of lamellar body contents were observed in human skin *ex vivo* (Kumamoto et al., 2013). Thus, we speculated that exocytosis from both neurons and keratinocytes might be regulated by the electrochemical status of these cell membranes.

Because permeability homeostasis is linked intimately to calcium dynamics, we assessed whether voltage-gated, calcium channels (VGCC) are functionally expressed and active in epidermis. Indeed, topical applications of two VGCC antagonists, nifedipine and verapamil, accelerated barrier recovery kinetics, while in contrast, applications of a VGCC activator, (S)-(−)-BAY K8644, delayed recovery in mouse epidermis (Denda et al., 2006). These studies are consistent with a prior study, which demonstrated that topical applications of calcium chloride delay barrier recovery, while co-applications of two VGCC blockers, verapamil and nifedipine, reverse the delay (Lee et al., 1992). We speculated that VGCC antagonists might block the expected increases in intracellular calcium ion and accelerate lamellar body exocytosis (Denda et al., 2006; Kumamoto et al., 2013). Thus, VGCC could serve as the key keratinocyte sensor of electric potential.

### 2.3 Visible radiation

Visible radiation occupies electromagnetic wavelengths between the ultraviolet and infrared. Because both ultraviolet and infrared radiation impact skin function (Rijken and Bruijnzeel, 2009), we hypothesized that intermediate wavelengths of visible light could also influence permeability barrier homeostasis. When we compared the impact of different wavelengths of colored light on barrier recovery, red light exposure accelerated, but blue light delayed barrier recovery, while green and white light exerted no influence. Accordingly, red light accelerated lamellar body secretion into the interstices between the stratum corneum and stratum granulosum, while blue light inhibited organelle secretion in hairless mouse epidermis (Denda and Fuziwara, 2008). Notably, Nishizawa and colleagues subsequently demonstrated that red light blocks alterations in skin surface electric potential after acute barrier disruption (Abe et al., 2019). Finally, red light also increases mitochondrial activity and epidermal proliferation (Umino and Denda, 2023).

We next asked whether the epidermis expresses the same types of visual receptors that are found in the retina, where rhodopsin senses brightness vs. darkness, while opsins distinguish red, green, and blue colors. Indeed, all known types of opsins are expressed in human keratinocytes (Tsutsumi et al., 2009a; Suh et al., 2020). Activation of opsins in the retina leads to electrochemical signaling by transducin and phosphodiesterase, while pertinently, an inhibitor of phosphodiesterase blocked the positive impact of red light on barrier recovery in mouse epidermis (Goto et al., 2011).

Another signal transduction cascade occurs in *drosophila* (Randall et al., 2015). In this case, signals from certain opsins activated phospholipase C followed by PIP2 formation, and TRP channel opening, while in contrast other known opsins trigger hyperpolarization. This cascade could also exist in the epidermal keratinocytes.

Recent studies elucidated a variety of effects of visible light on epidermis (Pierfelice et al., 2023; Pourang et al., 2022; Sutterby et al., 2022; Brown et al., 2024), suggesting that epidermal keratinocytes deploy a sensory system for visible radiation that functions similarly to the retina, though the details of its operation remain uncertain. Because ultraviolet (UV) radiation displays much stronger energy than visible radiation, it could damage keratinocyte metabolism in a variety of ways. Accordingly, a recent study demonstrated that UVA induces keratinocyte supranuclear melanin cap formation via opsin 3 (Lan et al., 2023). Another study demonstrated that toll-like receptor 3 can sense self-RNA released from necrotic keratinocytes following UV damage (Borkowski et al., 2015), further indicating that keratinocytes deploy sensory systems that protects the epidermis from damage induced by UV radiation.

Slominski and his co-workers demonstrated that UV radiation triggers local responses secondary to the induction of chemical, hormonal, immune, and neural signals that are defined by epidermal chromophores. These signals reach the brain, endocrine, and immune systems, as well as other central organs, which in concert regulate body homeostasis. Thus, these authors concluded that photo-neuro-immunoendocrinology can offer novel therapeutic approaches for psychological, autoimmune, neurodegenerative, endocrinological disorders (Slominski et al., 2018; Slominski et al., 2024).

A variety of photoreceptors are expressed in the epidermal keratinocytes and visible radiation and UV radiation influenced pathophysiology of the skin. Studies of this field could prove important for clinical dermatology.

### 2.4 Sound

We paraphrase an age-old question here: ‘Can the skin hear a tree fall in the forest?’. Indeed, Oohashi et al. (2006) illuminated the impact of completely inaudible, high-frequency sounds (hypersonic effects) on the human brain and endocrine system, suggesting further that epidermis could be the sensor of these sounds (Kawai et al., 2022). To address this possibility, we evaluated the impact of sound on permeability barrier homeostasis. While sub-detectible, 5 kHz sounds did not influence recovery rates, low register 10, 20, and 30 kHz sounds accelerated barrier recovery, accompanied by enhanced lamellar body secretion in mouse epidermis (Denda and Nakatani, 2010). Interestingly, hair growth, too, was induced by inaudible sounds (Choi et al., 2022). These results strongly suggest that epidermal keratinocytes possess a still uncharacterized sensory system that recognizes sounds above 10 kHz.

Though the receptors for sound in keratinocytes remain uncertain, one emergent candidate could be Piezo1 (Liao et al., 2019), consistent with its expression in keratinocytes (Mikesell et al., 2022). Pertinently, a recent study demonstrated that Piezo1 and Piezo2 might construct a mechano-sensitive complex in inner ear hair cells (Lee et al., 2024). Further studies on the role of both Piezo1 and Piezo2 could clarify the mechanisms by which the epidermis detects sound.

Recent report demonstrated that the benefits of activation of TRPV4 channels by low intensity ultrasound on knee osteoarthritis in mice (Wu et al., 2024). Another study indicated that PM 2.5 pollutants inhibit the growth of cilia in both epidermal keratinocytes and retinal pigment epithelium cells (Bae et al., 2019). These reports suggested that primary cilia in the keratocytes might serve as another sensory system for ultrasound in epidermis.

### 2.5 Pressure

We previously demonstrated that mechanical stimulation of keratinocyte monolayer cultures with a glass micropipette induces elevations in intracellular calcium, as well as calcium ion propagation via gap junctions and ATP receptors in human keratinocytes (Tsutsumi et al., 2009b). Accordingly, we compared changes in intracellular calcium levels produced in response to 25, 50, and 100 hPa hydraulic pressure in human keratinocytes (Goto et al., 2010). In response to 100 hPa pressure, elevations in intracellular calcium occur in both undifferentiated and differentiated keratinocytes, but lower pressures (25 or 50 hPa) only stimulated calcium levels in differentiated human keratinocytes (Goto et al., 2010). As described above, mechano-sensitive receptor, Piezo1 was expressed in keratinocytes (Mikesell et al., 2022). These results suggest that Piezo and/or a TRP-like, calcium ion channel could regulate the sensation of hydraulic pressure, and that keratinocytes in the upper viable layers of the epidermis could respond to changes in mechanical stress (Chien and Tsai, 2023).

### 2.6 Humidity

The impact of changes in environmental humidity on the skin have been investigated for over 2 decades. Because the stratum corneum becomes thicker and barrier recovery accelerates in a dry environment in mouse epidermis (Denda et al., 1998a), these changes could reflect helpful adaptations to arid environmental conditions. However, when hairless mice were shifted *in extremis* from an extremely dry to a humid environment, barrier function was temporarily compromised due to shedding of all suprabasal layers of the epidermis (Sato et al., 2002), paralleled by a decline in filaggrin levels in mouse epidermis (Katagiri et al., 2003). Thus, while barrier function does not adjust quickly to drastic reductions in humidity, more gradual reductions in external humidity stimulate concurrent improvements in barrier homeostasis. Notably, \such a reduced humidity also drives protease-driven hydrolysis of filaggrin into its constituent amino acids, followed by their deimination into hygroscopic polycarboxylic acids that enhance stratum corneum hydration, while also generating trans-urocanic acid, the principle UVB photophore in human stratum corneum (Moodycliffe et al., 1996).

Prolonged reductions in external humidity place additional stress on the permeability barrier that command an appropriate response. Cytokines represent one class of candidates that could respond. Because IL-1α is known to stimulate epidermal lipid synthesis (Barland et al., 2004), we hypothesized and then demonstrated that not only IL-1α levels increase after the mouse epidermis is exposed to a dry environment (Ashida et al., 2001b), but also mRNA levels of three other pro-inflammatory mediators, TNFα, IL-1β, and GM-CSF also increase (Wood et al., 1992; Elias et al., 1999). Indeed, under dry conditions even minor skin perturbations can provoke significant inflammation in mouse epidermis (Denda et al., 1998b; Denda, 2001). Hence, cytokine production and/or release could be stimulated by environmental aridity.

In our previous experiments using epidermal organotypic culture system, exposure to environmental dry condition increased cortisol secretion and mRNA levels of cortisol-synthesizing enzyme (steroid 11b-hydroxylase, CYP11B1) and IL-1β (Takei et al., 2013).

Pertinently, allergic reactions are amplified in a dry environment, and Langerhans cell densities increase under such dry conditions, again consistent with a more pronounced inflammatory response in mouse epidermis (Hosoi et al., 2000). Because TRPV4 activation and expression increase in corneal epithelia following exposure to hypotonic solutions (simulating dry conditions) (Lapajne et al., 2020), TRPV4, a known sensor of changes in osmotic pressure (Galindo-Villegas et al., 2016), could serve as the key humidity sensor in epidermis.

We previously demonstrated that exposure of cultured human keratinocytes to air increased intracellular calcium concentration and secretion of ATP. When we removed calcium from the medium or applied suramin, a purinergic receptor antagonist, reduced the increase of intracellular calcium (Denda and Denda, 2007). We also demonstrated that application of ATP induced IL-6 expression and secretion from cultured human keratinocytes (Inoue et al., 2007). Another report showed that following ATP stimulation, IL-1β is also released from keratinocytes and might induce inflammation (Burnstock and Knight, 2018). Those studies suggested that ATP might play a crucial role in inflammatory mechanisms induced by environmental dry conditions.

## 3 Chemical factors

### 3.1 Odorants

A variety of olfactory receptors (OR) have been identified in keratinocytes during the past decade. Previous studies demonstrated that activation of OR2AT4 and OR51B5 accelerates wound healing, and that activation of OR2A4/7 is linked to keratinocyte proliferation (Busse et al., 2014; Tsai et al., 2017). Moreover, applications of Sandalore®, a synthetic sandalwood odorant increased OR2AT4 expression in human skin organ cultures and induced dermcidin synthesis in the epidermal keratinocytes (Edelkamp et al., 2023). Sandalore® is an agonist of the cutaneous olfactory receptor OR2AT4. It induces strong Ca^2+^ signals in cultured human keratinocytes (Busse et al., 2014). Another study demonstrated that activation of OR2AT4 in human hair follicle epithelium prolonged hair growth (Cheret et al., 2018).

We recently found that OR5P2, OR5P3, and OR10A6 are also expressed in human keratinocytes, and that activation of OR10A6 accelerates terminal differentiation (Nakanishi et al., 2023). Moreover, UV exposure downregulated OR expression (Kang et al., 2021). These studies suggested that ORs might play important roles in a variety of aspects of epidermal homeostasis.

On the other hand, we recently demonstrated that odorant molecules could influence human keratinocyte metabolism not only via receptors, but also by changes in cell membrane conformation. Accordingly, trans-2-nonenal (2 TN) induces physiologic apoptosis in cultured keratinocytes, while ‘masking’ odorants, benzaldehyde and 4-anisaldehyde, rescued cells from 2 TN-induced apoptosis (Nakanishi et al., 2021). Rather than binding to olfactory receptors, these interactions reflect changes in cell membrane conformity in the olfactory epithelium, as detailed further below.

It has long been recognized that odorant receptors play a crucial role in molecular recognition in all living systems. Yet, how keratinocytes recognize and respond to odorants will remain elusive until their receptors have been cloned. Although the mechanism of human perception of odorant molecules remains only partially characterized, further studies of odorant sensory activation within cell membranes could open a new paradigm for our overall perception of volatile molecules.

### 3.2 Tastants

Bitter taste receptors, TAS2Rs, are expressed in epidermal keratinocytes (Shaw et al., 2018). Keratinocytes express the bitter taste receptors TAS2R1 and TAS2R38 promotes keratinocyte differentiation. Moreover, Amarogentin, an agonist for TAS2R1 and other TAS2Rs, reduces histamine-induced IL-8 and MMP-1 secretion (Wölfle et al., 2015). Among these, a TAS2R14 ligand induced an increase in intracellular free Ca^2+^ concentrations (Ho et al., 2021). TAS2R16 and TAS2R10 are expressed in HaCaT cells and regulate wound healing in aged HaCaT cell monolayers (Chung et al., 2022). A recent study demonstrated that activation of TAS2R38 leads to production of ABC transporters (Mori et al., 2024). The authors of this report suggest that TAS2Rs in the keratinocytes could facilitate the excretion of harmful molecules via ABCB1. Another recent report described a decrease of TAS1R3 in tape-stripped skin samples from children with allergic asthma (Del Duca et al., 2024).

A broader array of taste receptors could still be found in keratinocytes, which could in turn influence epidermal homeostasis.

### 3.3 Hormones and other small molecules

Epidermal keratinocytes express a variety of hormone receptors and some of these hormones influence epidermal pathophysiology. For example, application of melatonin downregulated the intraepidermal activity of the aging-promoting mTORC1pathway and MMP-1 protein expression (Samra et al., 2023). Slominski and his coworkers suggested that melatonin and some of its metabolites inhibit melanogenesis. Moreover, melatonin also accumulates in melanocytes where its antioxidative effects could stimulate the synthesis and activity of ROS scavenging enzymes and other antioxidants, while also promoting DNA repair, and enhancing mitochondrial function (Sevilla et al., 2022).

CYP11A1, a member of the cytochrome P450 family, generated in epidermal keratinocytes, plays several critical roles in the skin through its initiation of local steroidogenesis and specific metabolism of vitamin D, lumisterol, and 7-dehydrocholesterol. Products of these pathways regulate the protective barrier and skin immune functions in a context-dependent fashion through interactions with a large number of receptors (Slominski et al., 2021; Evendt et al., 2025).

Though the skin synthesizes and secretes a variety of hormones (Slominski et al., 2022), prior studies have demonstrated that among these hormones, testosterone and estrogen exert opposing effects on epidermal barrier function (Hanley et al., 1996). Examples of the negative effects of testosterone include: 1) Barrier recovery kinetics are delayed in adult vs. juvenile hairless mice (Kao et al., 2001); 2) Blockade of testosterone production with finasteride accelerates barrier recovery (Kao et al., 2001); 3) Epidermal lipid production is reduced in male vs. female hairless mice (Feingold et al., 1983); 4) Topical beta-estradiol enhances epidermal functions likely by increasing ceramide synthesis (Kendall et al., 2022). 5) In a patient receiving testosterone replacement by bi-monthly injections, barrier function declined immediately after injections, but returned back to baseline just prior to the next injection (Kao et al., 2001); and 6) while androgens (testosterone or androsterone) delayed barrier recovery, their impact could be countered by co-applications of beta-estradiol in mouse epidermis (Tsutsumi and Denda, 2007).

Because these phenomena occurred within 30 min after acute barrier disruption (+/- hormone applications), we suspected that these changes likely reflect interactions between these hormones and target cell membranes, achieved through a series of physicochemical changes rather than by more time-consuming genomic phenomena. Hence, we evaluated their impact on surface monolayers of 1,2-di-O-myristoyl-sn-glycero-3-phosphocholine (DMPC). While the surface pressure (π) isotherm for the monolayer increased in the presence of β-estradiol, testosterone had no effect (Nakata et al., 2011). Together, these results suggest that testosterone perturbs cell membranes, while β-estradiol exerts beneficial effects, paralleling their known impact on permeability homeostasis (Table 2).

**TABLE 2:** Physicochemical studies using phospholipid monolayers.

Test molecule	Phospholipid	Surface Pressure (π)	Impact on Barrier Recovery/Differentiation
Testosterone (Nakata et al. 2011)	DMPC	No effect	Delays recovery (Denda and Denda, 2007) 10 mM
Beta-estradiol (ibid)	DMPC	Increase	Blocks the effect of testosterone (ibid) 10 mM
Mannose, fructose (Nakata et al. 2012)	DOPC	Increase	Accelerates recovery (Denda, 2011) 0.1 M
Galactose, glucose (ibid)	DOPC	No effect	No effect (ibid) 0.1 M
Oleic acid (Nakata et al. 2017)	DOPC	Decrease	Barrier dysfunction (Katsuta et al., 2005) 10 mg/ml
Stearic acid (ibid)	DOPC	No effect	No effect (ibid) 10 mg/ml
2-trans-nonenal (Fujita et al. 2022)	DOPC	Increase	Keratinocyte apoptosis (Nakanishi et al., 2021) 50 mM
2-trans-nonenal	DOPC	No effect	No effect (ibid)
+ masking odorants (ibid)			50 mM

DMPC, 1,2-di-O-myristoyl-sn-glycero-3-phosphocholine.

DOPC, 1,2-dioleoyl-sn-glycero-3-phosphocholine.

Pertinently, molecules that influence phospholipid membrane phase transitions also impact lipid lamellar structures in differentiated keratinocytes as well as exocytosis of pro-lamellar lipids during epidermal terminal differentiation in human skin *ex vivo* (Umino et al., 2019; Denda et al., 2020). Thus, the effects of sex hormones on barrier homeostasis could reflect interactions between hormones and cell membranes.

We next evaluated the effects of added saturated and unsaturated free fatty acids on cultured keratinocytes, and in parallel, on permeability barrier homeostasis. Only unsaturated fatty acids induced intracellular calcium elevation and barrier dysfunction (Katsuta et al., 2005) (Table 2). Pertinently, the (π) isotherm for 1,2-di-stearoyl-sn-glycero-3-phosphocholine (DOPC monolayers declined after addition of oleic acid, while addition of stearic acid exerted no impact (Nakata et al., 2017) (Table 2).

As described above, 2-trans-nonenal (2 TN) induced apoptosis of cultured human keratinocytes, while two ‘masking’ odorants, blocked 2 TN-induced apoptosis (Nakanishi et al., 2021). The surface pressure of such DOPC monolayers increased upon the addition of 2TN, while the ‘masking’ odorants blocked the expected increase (Fujita et al., 2022) (Table 2). This series of studies suggest that phospholipid-enriched monolayers, as models of cell membranes, respond differently to volatile molecules in parallel to their known impact on barrier function (Denda et al., 2020).

A variety of hormones synthesized and released from epidermal keratinocytes could influence epidermal homeostasis and whole-body physiology, including psychological conditions (Denda et al., 2013). To clarify the effects of keratinocytes derived hormones, keratinocytes-specific, conditional knock-out animal study would be required.

## 4 Additional biological factors relevant to barrier homeostasis

1. Toll-like receptors (TLRs), also called ‘alarmins’ (Gallo and Nakatsuji, 2011), were originally found in immune cells, such as macrophages and dendric cells, where they distinguish characteristic structures of bacteria and viruses. But TLR 3, 4, 5, and 9 also are functionally expressed in keratinocytes (Lebre et al., 2007), and activation of TLR3 is required for barrier recovery following UVB-induced damage (Borkowski and Gallo, 2014; Borkowski et al., 2015). Thus, keratinocytes should be included, along with Langerhans cells, as outermost guardians of the cutaneous immune system.
2. Protease activated receptors: Four types of protease-activated receptors (PAR) are expressed in epidermis; i.e., PAR-1, PAR-2, PAR-3, and PAR-4. While thrombin activates PAR1, PAR-2 is activated by trypsin (kallikreins) (Rattenholl and Steinhoff, 2008). Previous studies demonstrated that PAR2 expression in keratinocytes regulates both epidermal barrier homeostasis and epidermal terminal differentiation in human and hairless mice skin (‘physiological apoptosis’) (Hachem et al., 2006). Moreover, mite and cockroach allergens display protease activity and are capable of disrupting barrier function in human and hairless mice skin (Jeong et al., 2008). Accordingly, we demonstrated that Japanese cedar pollen allergen (Cry J1) activates protease activity in keratinocytes, leading to a PAR1-mediated compromise in barrier homeostasis in human skin *ex vivo* (Kumamoto et al., 2016; Nakanishi et al., 2018). In each case, elevations in intracellular calcium ions were observed, which in turn could be blocked by calcium channel blockers and calmodulin antagonists in hairless mouse skin (Lee et al., 1992).

## 5 Regulation of barrier homeostasis, desquamation, and inflammation by alterations in surface pH

**FIGURE 1:**
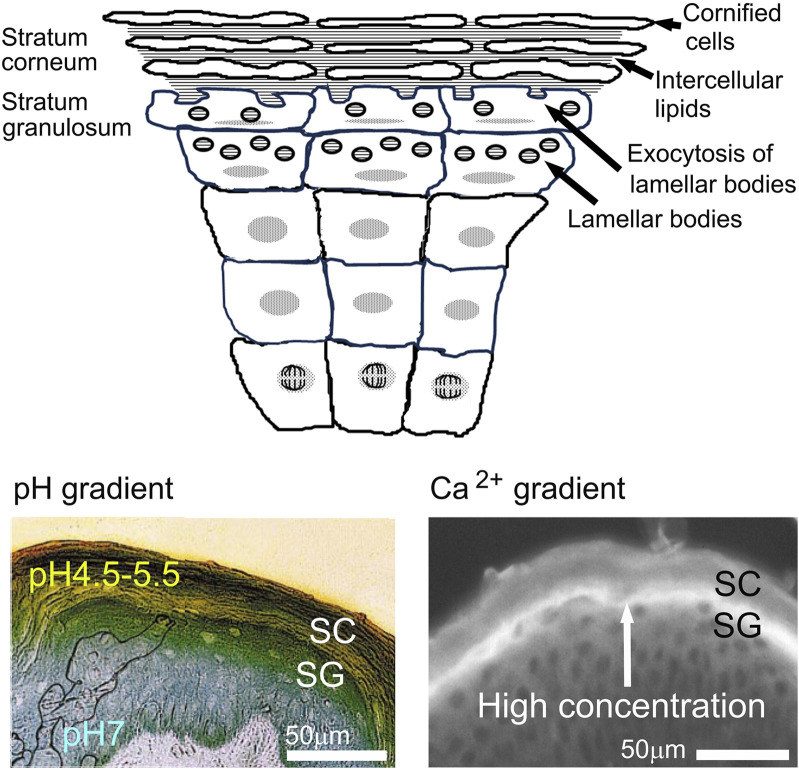
Schematic illustration of the epidermal water-impermeable barrier and visual images of pH and calcium ion gradations in the epidermis [modified from Denda et al. (2000); Hatano and Elias (2023)].

Consider next that the skin surface exhibits an extremely low surface pH (4.5-5.0), with the lowest levels observed in deeply pigmented skin (Gunathilake et al., 2009; Hatano and Elias, 2023; Brooks et al., 2024) (Figure 1). The functional implications of a reduced pH are manifold. First, the reduced pH of deeply pigmented skin accounts for its functional superiority (see below). Second, several kallikreins (serine proteases) that regulate the shedding of corneocytes (and conversely its cohesion) require a neutral-to-alkaline pH to be activated. Hence, an acidic surface pH slows desquamation rates (Hachem et al., 2003; Hachem et al., 2005). Third, the acidic surface of the stratum corneum is well known to inhibit the growth of pathogenic staphylococci and streptococci, while conversely, the risk of colonization by these pathogens increases at the elevated surface pH that characterizes inflammatory dermatoses. Fourth, the two enzymes that regulate the generation of ceramides from their immediate precursors (i.e., β-glucocerebrosidase and acidic sphingomyelinase) require an acidic surface pH (Takagi et al., 1999). Finally, the same neutral pH-requiring kallikreins that regulate desquamation also can initiate inflammation by converting corneocyte reservoirs of the pro-forms of epidermal IL-1α & β into their active, pro-inflammatory products.

But the ‘pH story’ does not end there–keratinocytes express plasminogen activator receptors type 2 (PAR2) on their surface, which also are activated by kallikreins. PAR2 in turn triggers terminal differentiation (physiologic apoptosis) leading to stratum corneum formation, while inhibitors of PAR2 delay barrier recovery after acute abrogations (Demerjian et al., 2008). Together, these studies illuminate the important roles of acidification for epidermal homeostasis (Fluhr and Elias, 2002).

A recent report demonstrated that an acid-sensitive ion channel (ASIC1a) was expressed in airway epithelial cells and inhibition of ASC1a reduced the pyroptosis induced by an extracellular acidic environment (Tan et al., 2024). TRPV1 in keratinocytes is also activated by low pH (Inoue et al., 2002). Hence, ASICs and/or TRPV1 might play an important role in the epidermal barrier homeostasis.

## 6 Keratinocyte-brain axis

### 6.1 Direct communication between keratinocytes and the peripheral nervous system

Because the epidermis and central nervous system arise in parallel from the primitive neuroectodermal layer that encases the early embryo, the epidermis has retained features typically associated with nervous tissues. Accordingly, we previously demonstrated that mechanical stimulation of human keratinocytes induced retrograde excitation of rat neurons (Tsutsumi et al., 2009b). When we applied apyrase, an ATP-degrading enzyme, excitation declined significantly, suggesting that ATP release from keratinocytes could mediate signal transfer between keratinocytes and the peripheral nervous system. On the other hand, because excitation was not completely abolished by apyrase, there could be additional types of communication between keratinocytes and the peripheral nervous system, including direct synaptic communication between keratinocytes and peripheral nerves (Talagas et al., 2020a; Talagas et al., 2020b; Xu et al., 2022).

A series of prior studies demonstrated that excitation of keratinocytes is recognized by the brain. First, Pang et al. (2015) demonstrated that keratinocyte stimulation by capsaicin, after prior binding to TRPV1, induced nociception-related responses. These researchers then developed a keratinocyte-specific, TRPV1 knockout mouse model, and observed that immediately after capsaicin applications, wild-type mice started paw-licking, while no such behavior was observed in the knockout mice.

Similarly, Baumbauer et al., 2015 established transgenic mice that express the light-sensitive protein, rhodopsin, in the epidermis. When these mice were exposed to otherwise harmless, visible light, they retreated as if in response to painful stimuli. These authors then demonstrated that either mechanical stress, heat, or laser stimulation of mouse skin induces electric responses in dorsal-root-ganglia.

#### 6.1.1 Potential mechanisms

Painful tactile stimuli (Moehring et al., 2018), UVB irradiation, acute barrier disruption (Denda et al., 2002a; Denda et al., 2002b; Inoue et al., 2007), as well as exposure to a reduced humidity all induce ATP release from keratinocytes, and the released ATP in turn activates the peripheral nervous system via the ATP receptor, P2X4 (Denda and Denda, 2007; Moehring et al., 2018). These results suggest that a variety of environmental stimuli stimulate ATP release from keratinocytes. Epidermal keratinocytes generate not only ATP but also a variety of other mediators, including dopamine and nitrous oxide that can impact the peripheral nervous and/or vascular systems in mouse skin (Fuziwara et al., 2005; Ikeyama et al., 2010). Furthermore, Sadler et al. (2020) demonstrated that both ATP from keratinocytes and P2X4 in the peripheral nervous system play an important role in cold and heat sensation. Finally, Talagas et al. (2020b) suggested that either prostaglandin E2 or endothelin-1 released from keratinocytes could be involved in cutaneous nociception (pain perceived from the skin). Together, these results suggest that epidermal keratinocytes mediate cutaneous sensation and CNS responses in response to a variety of environmental stimuli.

### 6.2 Endocrine factors released from keratinocytes appear to influence barrier homeostasis

When the epidermis is exposed to stressful, arid conditions, cortisol is generated and released from human epidermal keratinocytes as described above (Takei et al., 2013). Moreover, such conditions induce ATP release from keratinocytes, and ATP in turn induces IL-6 release from human keratinocytes via purinergic receptors (Inoue et al., 2007). Elevations of cortisol and cytokines, particularly IL-6, damage the hippocampus, potentially inducing anxiety or depression. Thus, factors that stress the epidermal barrier, such as xeric conditions, could impact human emotions, though further research is needed to illuminate this potential relationship.

Though not yet directly linked to barrier homeostasis, oxytocin (OT) is both generated by and sensed by human keratinocytes (Denda et al., 2012b). Non-invasive, tactile stimuli that increase plasma OT levels positively influence emotional status (Portnova et al., 2020), while also enhancing barrier function. Moreover, systemic OT infusions dampen repetitive behavior in patients with autism and Asperger’s syndrome (Hollander et al., 2003; Li et al., 2022), and improve wellbeing in war veterans suffering from post-traumatic stress (Eidelman-Rothman et al., 2015). Thus, OT generated and released from keratinocytes could play an important role in mediating the effects of tactile stimuli on both emotion and barrier function. Together, these studies suggest that a suite of sensory receptors in keratinocytes could influence human psychological status.

Slominski and his co-workers have published important reviews about the neuro-immuno-endocorinology of the skin. In these reviews, they suggest that environmental factors, including solar radiation, biological, physical and chemical insults, and pollutants, a variety of mediators, including pituitary and hypothalamic hormones, neuropeptides, cytokines and chemokines, biogenic amines, serotonin, melatonin, cannabinoids, steroids, and secosteroids are generated in the epidermal cells and regulate protective responses against environmental insults. Recently, they suggested that topical application of melatonin or its metabolites can be used to prevent and treat skin disorders and cutaneous aging (Slominski AT. et al., 2025). Moreover, this skin neuro–immuno–endocrine system communicates with the local microbiome, neural, endocrine and immune systems and regulate local and central homeostasis (Slominski and Wortsman, 2000; Slominski et al., 2022; Slominski RM. et al., 2025).

## 7 Neurotransmitters and barrier function

As described above, epidermal keratinocytes deploy a large complement of sensory receptors that detect potential environmental threats. This series of epidermal neurotransmitters could in turn play crucial roles in signaling the brain (Table 3). Pertinently, these receptors are not only functionally expressed in keratinocytes, but they also have been shown to influence permeability barrier homeostasis. Moreover, receptors for endogenous molecules like histamine and ryanodine are also expressed in keratinocytes and have been shown to impact permeability barrier function in mouse skin (Ashida et al., 2001a; Denda et al., 2012a; Lin et al., 2013) (Table 3). Keratinocytes also express nuclear-hormone receptors (Schmuth et al., 2008), as well as cannabinoid receptors (Roelandt et al., 2012), both of which are known to regulate epidermal barrier function, though by different mechanisms.

**TABLE 3:** Effects of receptor agonists and antagonists on skin permeability barrier recovery.

Neurotransmitter Receptors	Barrier Recovery Acceleration	Barrier Recovery Delay
Ionotropic Receptors		
P2X receptor (Denda et al., 2002a)	Antagonist TNP-ATP (100 nM)	Agonist α,β-methylene ATP (1 mM),
NMDA receptor (Fuziwara et al., 2003)	Antagonist MK-801 (1 mM)	Agonist NMDA (1 mM)
Cholinergic receptor (Denda et al., 2003b)	NYP	Agonist nicotine (1 mM)
GABA(A) receptor (Denda et al., 2002b)	Agonist GABA (100 μM)	NYP
Glycine receptor (Denda et al., 2003b)	Agonist Glycine (1 mM)	NYP
VGCC (Denda et al., 2006)	Antagonist nifedipine (1 mM)	Agonist S-(–)-BAY K8644 (1 mM)
Potassium channel (Denda et al., 2007b)	Agonist diazoxide (1 mM)	NYD
G-Protein coupled Receptors		
Adrenergic β2 receptor (Denda et al., 2003a)	Antagonist ICI-118 551 (1 mM)	Agonist procaterol (1 mM)
Dopamine 2-like receptor (Fuziwara et al., 2005)	Agonist bromocriptine (100 nM)	Antagonist L-741626 (100 nM)
Serotonin receptor (Denda, 2005b)	Agonist 5-Hydroxytryptamine (1 mM)	NYP
Endocrine Receptors		
Histamine receptor H1, H2 (Ashida et al., 2001a)	Antagonist diphenilhydoramine (5% wv)	NYP
Ryanodine receptor (Denda et al., 2012a)	Antagonist dantrolene (100 μM)	Agonist 4-chloro m-cresol (2.5 mM)

All barrier recovery studies were carried out using hairless mice. When we observed intracellular calcium ion dynamics, we used cultured human keratinocytes. All reagents were applied as aqueous solution.

## 8 Conclusion

That the epidermis deploys a broad suite of sensory functions should not be surprising from an evolutionary and developmental biology standpoint. Cnidarians, which are among the earliest multicellular organisms, express a series of sensory receptors, including rhodopsin and a neurotransmitter receptor that recognize NMDA (Watanabe et al., 2009). Because a scattered nervous system coats the surface of their bodies, most of these sensory receptors likely are expressed in their ‘skins.’

**FIGURE 2:**
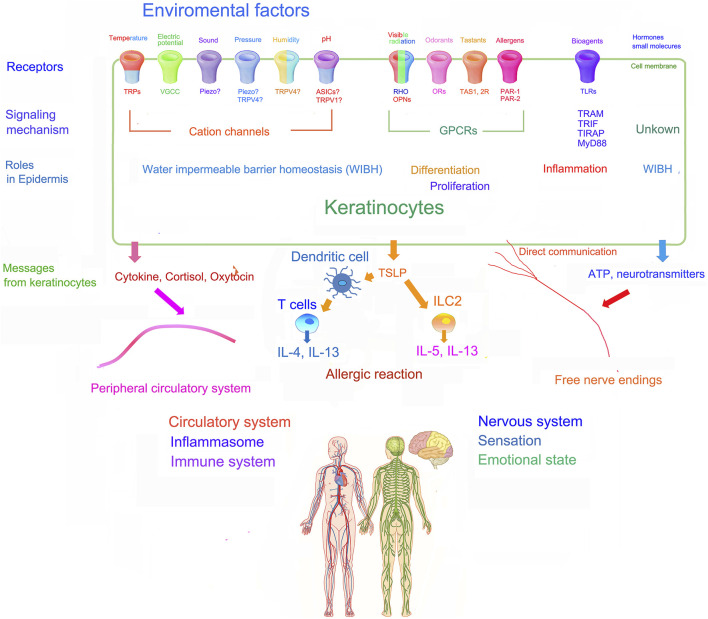
Schematic illustration of the role of sensory systems of epidermal keratinocytes and their potential influence on whole body physiology [modified from Tagalas et al. (2020b); Denda and Nakanishi (2022)].

During the earliest stages of human development, a primitive ectodermal layer coats the surface of the embryo, forming a neuroectodermal layer from which the central nervous develops, while leaving the remaining ectoderm to generate the epidermis. Thus, a variety of sensory receptors, as well as receptors for endogenous factors, like hormones and neurotransmitters, are co-expressed in the epidermis and central nervous system. Parts of various human sensory organs, including the eyes, ears, and nose also derive from this neuroectodermal layer. Odorants and taste sensations represent two deeply preserved sensory systems that have persevered throughout human evolution. Because epidermal keratinocytes express a full panoply of such sensory mechanisms, as well as the necessary information processing systems, awareness of the epidermis’ updated capabilities could lead to a new medical discipline that embraces a role for the skin’s powerful sensory systems in multiple aspects of psychological health (Figure 2).

## Abbreviations

4αPDD, 4alpha-phorbol 12,13-didecanoate; ATP, adenosine triphosphate; Ca^2^+, ionized calcium; Cry J1, Japanese cedar pollen allergen; DMPC, 1,2-Dimyristoyl-sn-glycero-3-phosphocholine; DOPC, 1,2-Dioleoyl-sn-glycero-3-phosphocholine; NMDA, N-methyl-D-aspartate; OR, olfactory receptor; OT, oxytocin; PAR, protease-activated receptor; TLR, toll-like receptor; TRP, transient receptor potential; TRPA, Transient receptor potential cation channel subfamily A; TRPV, transient receptor potential vanilloid; VGCC, voltage-gated calcium channel.
